# A High-Content Assay Enables the Automated Screening and Identification of Small Molecules with Specific ALDH1A1-Inhibitory Activity

**DOI:** 10.1371/journal.pone.0170937

**Published:** 2017-01-27

**Authors:** Adam Yasgar, Steven A. Titus, Yuhong Wang, Carina Danchik, Shyh-Ming Yang, Vasilis Vasiliou, Ajit Jadhav, David J. Maloney, Anton Simeonov, Natalia J. Martinez

**Affiliations:** 1 National Center for Advancing Translational Sciences, National Institutes of Health, Rockville, MD, United States of America; 2 Department of Environmental Health Sciences, Yale School of Public Health, New Haven, CT, United States of America; Columbia University, UNITED STATES

## Abstract

Aldehyde dehydrogenase enzymes (ALDHs) have a broad spectrum of biological activities through the oxidation of both endogenous and exogenous aldehydes. Increased expression of ALDH1A1 has been identified in a wide-range of human cancer stem cells and is associated with cancer relapse and poor prognosis, raising the potential of ALDH1A1 as a therapeutic target. To facilitate quantitative high-throughput screening (qHTS) campaigns for the discovery, characterization and structure-activity-relationship (SAR) studies of small molecule ALDH1A1 inhibitors with cellular activity, we show herein the miniaturization to 1536-well format and automation of a high-content cell-based ALDEFLUOR assay. We demonstrate the utility of this assay by generating dose-response curves on a comprehensive set of prior art inhibitors as well as hundreds of ALDH1A1 inhibitors synthesized in house. Finally, we established a screening paradigm using a pair of cell lines with low and high ALDH1A1 expression, respectively, to uncover novel cell-active ALDH1A1-specific inhibitors from a collection of over 1,000 small molecules.

## Introduction

The superfamily of human Aldehyde dehydrogenase (ALDH) enzymes comprises 19 putative functional isozymes that catalyze the NAD(P)+-dependent oxidation of an aldehyde to its corresponding carboxylic acid [1, 2]. ALDHs have a surprisingly broad spectrum of biological activities through the metabolism of both endogenous and exogenous aldehydes. For instance, they are involved in the biosynthesis and metabolism of the developmental regulator retinoic acid and the neurotransmitters GABA and dopamine, as well as in cellular homeostasis via the elimination of reactive aldehydes that arise as by-products of oxidative stress[3–5]. From a therapeutic point of view, ALDH activity is important in alcohol metabolism through aldehyde detoxification and to cancer drug resistance through the metabolism of chemotherapeutics such as cyclophosphamides [3, 6–8]. We focused our efforts on ALDH1A1, which in conjunction with two other cytosolic isozymes, ALDH1A2 and ALDH1A3, comprise the ALDH1A subfamily. Unbalanced ALDH1A1 activity has been linked to insulin resistance, obesity and inflammation [9–12]. Additionally, increased expression and activity of ALDH1A1 has been identified in a wide-range of human cancer stem cells and are associated with cancer relapse and poor prognosis [13, 14]. Given the significant physiological and pathological roles of ALDH1A1, there has been an interest in the development of small molecule inhibitors, not only as chemical tools to better understand the biological role of this protein but also for potential clinical applications [15, 16].

To date, most of the high-throughput technologies supporting the identification of small molecule modulators of ALDH1A1 activity constitute *in vitro* biochemical assays which, although robust and sensitive, do not study the enzyme in its native cellular state nor provide information of inhibitor’s cell permeability and toxicity. The evident need for complementary cellular approaches was recently addressed by Ming et al., where the authors adapted the commercially available, low-throughput flow cytometry-based ALDEFLUOR assay into a medium-throughput (96-well) imaging-based assay to assess ALDH1A1 inhibitors in hepatocarcinoma cell lines[17]. While a valuable starting point, this assay format is still not suitable to assess the cellular activity of compound libraries of >100 molecules in dose-response typically required to support systematic and thorough medicinal chemistry efforts.

Here, we have optimized, fully automated, and miniaturized a 1,536-well high-content ALDEFLUOR assay suitable to support quantitative high-throughput screening (qHTS) for the discovery, characterization and profiling of ALDH1A1 small molecule inhibitors. We show robust and reproducible assay performance in 5 μL volume and demonstrate the utility of this assay by generating 11- and 16-point dose response curves on a comprehensive set of prior art inhibitors (Validation Set), as well as an in-house library of over 300 proprietary ALDH1A1 inhibitor analogs, in cell lines displaying different ALDH expression levels. Finally, we implemented a dual cell-based phenotypic screening paradigm to directly uncover novel and selective ALDH1A1 inhibitors with cellular activity from large compound collections, a process that bypasses the initial biochemical screen and subsequent counterscreens for target specificity.

## Results

### Miniaturization and optimization of a 1,536-well imaging-based ALDEFLUOR assay

The ALDEFLUOR assay is used to identify and isolate living cells on the basis of ALDH activity. This assay takes advantage of the conversion of the fluorescent ALDH substrate BODIPY-aminoacetaldehyde (BAAA), which freely diffuses in and out of cells, into the negatively charged product BODIPY-aminoacetate (BAA), which is specifically retained inside cells thereby enhancing their fluorescence[18]. Although primarily dependent on ALDH1A1, the ALDEFLUOR assay reportedly detects activity from other subfamily members, namely ALDH1A2 and ALDH1A3, as well as mitochondrial ALDH2 [13, 19, 20]. 4-*N*,*N*-diethylaminobenzaldehyde (DEAB), a compound that inhibits multiple ALDH family members but displays the highest potency against 1A1, is frequently used as control for the assay[21]. To support our screening campaign for the identification of novel ALDH1A1 inhibitors for oncology research as well as other applications, we first sought to miniaturize and automate the previously established 96-well imaging-based ALDEFLUOR assay by Ming, et al.[17], to a 1,536-well format that could be implemented on an online robotic screening system to assess the cellular activity of thousands of ALDH1A1 inhibitors in a dose-response manner (qHTS).

**Fig 1:**
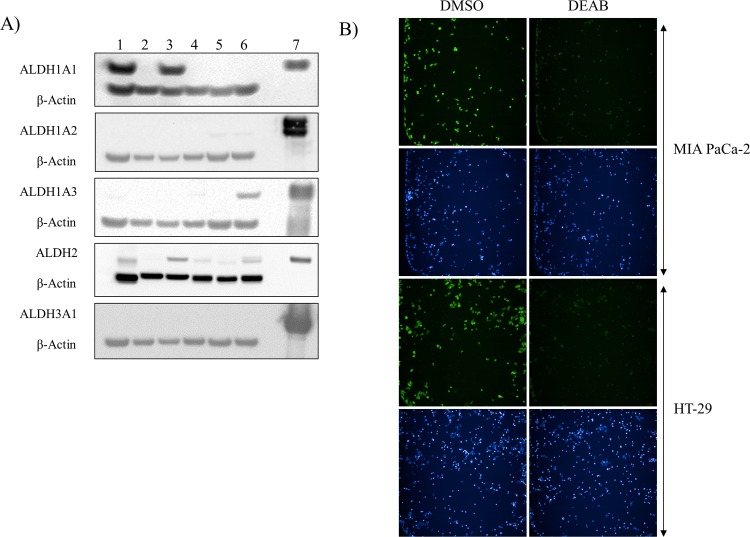
ALDH1A1 is differentially expressed in cancer cell lines and its activity can be indirectly visualized by BAA fluorescence. (**A**) Protein extracts from pancreatic cancer cell lines MIA PaCa-2 (lane 1) and PANC-1 (lane 2), colon cancer cell lines HT-29 (lane 3) and SW480 (lane 4) and glioma cell lines LN-18 (lane 5) and LN-229 (lane 6) were analyzed by immunoblot for ALDH1A1, ALDH1A2, ALDH1A3, ALDH2 and ALDH3A1 expression. β-Actin was used as loading control. The corresponding recombinant protein was loaded for reference (lane 7). (**B**) Representative fluorescent images of MIA PaCa-2 and HT-29 cells incubated with 500 nM of BAAA substrate and treated with 22 μM DEAB or DMSO for 30 minutes. Green fluorescent images indicate intracellular BAA and blue fluorescent images indicate nuclei staining.

High expression of ALDH1A1 has been reported in several tumor and cancer cell lines [13, 15]. To establish our assay, we selected a pair of cell lines from three different human cancer types, each pair consisting of a cell line with high and low reported ALDH1A1 levels[22–24]. Specifically, we tested glioma (LN-18 and LN-229), pancreatic (MIA PaCa-2 and PANC-1) and colon cancer cell lines (HT-29 and SW480). We confirmed by Western blotting that both MIA PaCa-2 and HT-29 express high levels of ALDH1A1, while their counterparts PANC-1 and SW480, present undetectable ALDH1A1 protein levels (Fig 1A). However, we were not able to detect significant ALDH1A1 expression in either glioma cell line, even though earlier reports indicated that LN-18 had high ALDH1A1 protein levels[22]. Our Western blot analysis also shows that both glioma cell lines express low levels of ALDH1A2; ALDH1A3 is mainly expressed in LN-229; and ALDH3A1 protein was not detected in any of the cell lysates (Fig 1A). ALDH2 expression was detected in all cell lines, with the exception of PANC-1. However, we should bring into question the specificity of the ALDH2 antibody used in this study. Our antibody cross-reactivity analysis shown in S1 Fig indicates that this particular ALDH2 antibody can recognize multiple ALDH isozymes. Limited cross-reactivity was seen for the remaining antibodies.

To miniaturize the assay into a 1536-well format amenable to qHTS, we chose MIA PaCa-2 and HT-29 because of their high levels of ALDH1A1(Fig 1A). Utilizing the 8-step protocol described by Ming et al. as a starting point[17], we developed a “semi-automated” protocol described in S1 Table. Briefly, we plated 1,000 cells in a volume of 5 μL/well into black-optical quality clear bottom 1,536-well plates, and allowed cells to attach overnight. Culture media was subsequently removed by inverting and centrifuging plates using a plate adaptor as previously described [25] and replaced with 5 μL of a solution of either 500 or 100 nM BAAA substrate in ALDEFLUOR buffer. To identify cells based on nuclear staining, we also included 0.5 nM Hoechst 33342 in the above buffer. Twenty-three nL of DEAB or DMSO vehicle (final assay concentration of DMSO 0.5%) was transferred via Wako Pintool. After incubating the plate for 30 minutes at 37°C, 5% CO_2_, 85% RH, the remaining substrate was removed by centrifugation as above and replaced with 3 μL of ALDEFLUOR buffer. Fluorescence was subsequently imaged using the IN Cell Analyzer 2200 high-content widefield fluorescence based imager and the resulting images were quantified using the IN Cell Investigator v1.6.2 analysis software (see Experimental Procedures for analysis details). These initial experiments in 1,536-well format revealed that ALDH1A1 activity can be detected in cell lines expressing high levels of ALDH1A1 such as MIA PaCa-2 and HT-29, but not low-expressing lines such as LN-18 and PANC-1 cells (Fig 1B and S2A Fig). In addition, these experiments indicated that fluorescence intensity correlated with the substrate concentration used in the assay, since cells treated with 500 nM substrate display higher intensity in the FITC channel compared to those treated with 300, 100 and 50 nM substrate (S2B Fig).

**Fig 2:**
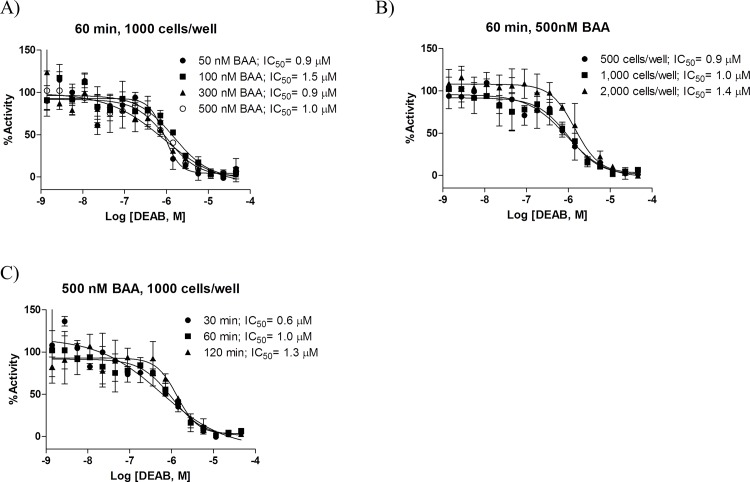
Assay miniaturization to 1,536-well format. **(A)** DEAB dose response curves of 1,000 MIA PaCa-2 cells/well incubated with the indicated concentration of BAAA substrate for 60 min. **(B)** DEAB dose response curves of indicated number of MIA PaCa-2 cells/well incubated with 500 nM of BAAA substrate for 60 min. **(C)** DEAB dose response curves of 1,000 MIA PaCa-2 cells/well incubated with the indicated concentration of BAAA substrate for 60 min. Data are represented as mean +/- SD, n = 3, and normalized to DMSO (100% activity) and 4.6 μM DEAB (0% activity).

**Fig 3:**
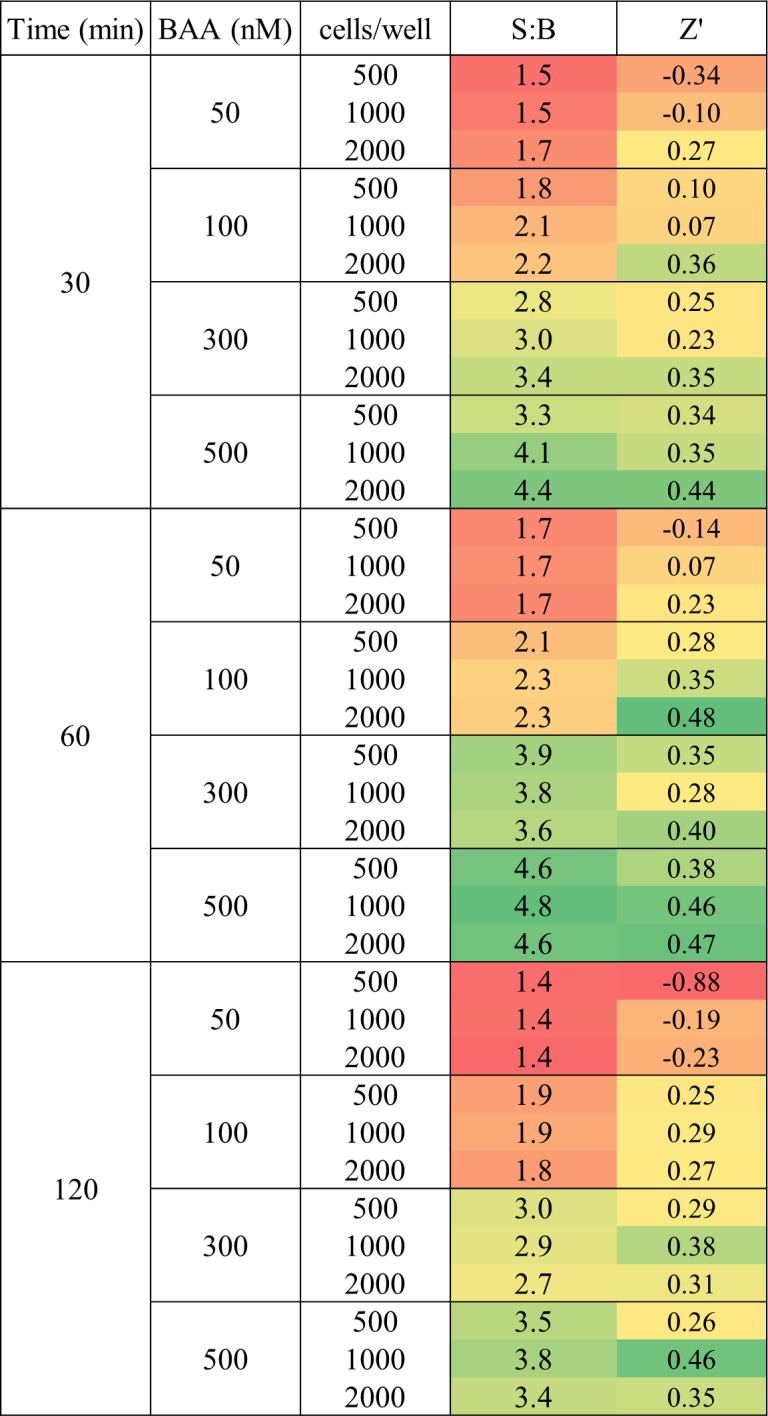
Assay optimization. S:B and Z’ factor heat map according to variation in assay parameters. Green represents best assay conditions. Parameters were calculated using signal from control DMSO wells and inhibitor DEAB (4.6 μM final) wells with n = 32.

To optimize the 1,536-well assay, we systematically tested assay signal-to-background (S:B) and robustness (Z’ factor) for three main parameters: BAAA substrate concentration (50–500 nM), number of MIA PaCa-2 cells (500–2,000 cells/well) and DEAB inhibitor incubation time (30–120 minutes). The observed DEAB’s IC_50_ increased with increasing cell numbers and also with longer reaction times. In contrast, no clear correlation was observed between DEAB’s IC50 and substrate concentration. Overall, the IC_50_ obtained for DEAB ranged from 0.6 to 1.5 μM with an MSR[26] of 2.2 (Fig 2A–2C). The best S:B and Z’ values were observed with higher number of cells/well and/or higher BAAA substrate concentrations (Fig 3). We found that inhibitor incubation time did not significantly influence assay performance, although incubation times of 30–60 minutes seem to be optimal for the cell line tested (Fig 3), and that it was crucial to remove background fluorescence due to remaining BAAA substrate in the supernatant before imaging (step 6 of semi-automated protocol in S1 Table). Although the IC_50_ for DEAB was similar with and without step 6, we found that removing background fluorescence due to the remaining BAAA substrate before imaging improves assay signal window and robustness, even when low substrate concentrations are used (S3 Fig).

### Validation set

To validate our optimized assay, we assembled a set of 20 previously reported ALDH inhibitors, with varying degrees of target specificity (Table 1 and S4 Fig) [15, 16, 27]. For instance, we included inhibitors like Bay-11-7085, Disulfiram and Aldi-2 because of their activity towards multiple ALDH isoforms, and inhibitors like NCT-501 and A37 with reported selectivity towards ALDH1A1. We also mined the patent literature to find three previously unpublished ALDH1A1 inhibitors, UM 673A, UM 673B, and Compound 5. In addition, we included two ALDH1A1 inhibitors (PubChem CID 725345 and 2929292) previously identified from our screening efforts (PubChem AID 1030). Furthermore, we incorporated Daidzin and CVT-10216, which preferentially inhibit ALDH2, and CB7 which specifically targets ALDH3A1, to ensure coverage of other ALDH isoforms. We initially set out to confirm the reported potency and specificity of this validated set of inhibitors using an *in vitro* fluorescence-based enzymatic assay [28]. Briefly, the assay measures the dehydrogenase activity of recombinant human ALDH1A1, ALDH1A2, ALDH1A3, ALDH2 or ALDH3A1 using NAD(P)^+^ and either propionaldehyde or benzaldehyde as substrates. An orthogonal resorufin-based assay format was also run with isolated ALDH1A1 to rule out potential false positives due to intrinsic compound fluorescence [29]. Both assay formats yielded comparable IC_50_ for each inhibitor (R^2^ = 0.86; S5A Fig).

**Table 1:** IC_50_ values of validation set compounds in biochemical and cell-based assays.

				Biochemical IC_50_ [μM]					Aldefluor IC_50_ [μM]	
Compound	Reported ALDH Target	Reported Biochemical IC_50_ [μM]	Reference	ALDH1A1	ALDH1A2	ALDH1A3	ALDH2	ALDH3A1	MIA PaCa-2	HT-29
indoline-2,3-dione	1A1	0.02	^[^^30^^]^	0.01	0.68	7.62	21.47	2.41	12.04	17.77
NCT-501	1A1	0.04	^[^^28^^]^	0.05	21.47	13.55	24.09	ND	4.14	4.33
CID 2929292	1A1	0.09	^[^^31^^];[^^28^^]^	0.29	3.03	24.09	9.59	21.47	ND	ND
Compound 5	1A1	0.39	^[^^32^^]^	0.49	21.47	21.47	24.09	24.09	1.01	0.79
Pargyline	1A1	1.6	^[^^16^^]^	30.33	24.09	17.05	ND	ND	ND	33.50
CID 725345	1A1	3.5	^[^^31^^];[^^28^^]^	0.77	0.34	3.82	2.15	5.39	9.46	14.47
A37	1A1	4.6	^[^^33^^]^	19.13	21.47	24.09	21.47	ND	ND	ND
Gossypol	1A1	75	^[^^16^^]^	25.56	5.39	8.55	24.09	27.03	ND	16.51
Kynurenine	1A1	NA	^[^^16^^]^	40.51	24.09	27.03	ND	ND	ND	ND
Molinate	1A1	NA	^[^^16^^]^	27.03	ND	ND	ND	ND	ND	ND
Citral	1A1	NA	^[^^16^^]^	10.76	ND	2.70	4.28	ND	27.77	26.61
Disulfiram	1A1; 2	0.15; 1.45	^[^^34^^];[^^35^^]^	0.03	0.14	0.68	0.76	1.91	10.95	10.87
CVT-10216	1A1; 2	1.3; 0.029	^[^^35^^];[^^36^^];[^^37^^]^	19.13	0.34	27.03	1.21	17.05	ND	ND
UM 673A	1A1, 1A2, 1A3	0.22; 0.21; 0.17	^[^^27^^];[^^38^^]^	7.02	NT	NT	36.44	NT	ND	ND
Bay-11-7085	1A1; 2; 3A1	0.074;0.034; 0.1	^[^^31^^];[^^28^^]^	0.09	0.38	0.68	0.05	0.54	20.03	14.34
Aldi-2	1A1; 2; 3A1	2.5; 6.4; 1.9	^[^^39^^]^	0.15	0.12	0.24	0.10	0.04	18.02	17.54
DEAB	1A1; 1A2; 1A3; 2	0.057; 1.2; 3.0; 0.16	^[^^21^^]^	0.08	3.40	24.09	8.55	6.05	3.44	1.69
UM 673B	1A1, 1A2, 1A3, 2	0.45; 0.8; 1.3; 1.7	^[^^27^^];[^^38^^]^	0.87	NT	NT	2.54	NT	20.72	11.36
Daidzin	2	0.29	^[^^40^^];[^^41^^]^	ND	21.47	24.09	21.47	ND	ND	ND
CB7	3A1	0.2	^[^^42^^]^	ND	15.20	12.07	19.13	0.38	ND	ND

ND: Not determined in the concentration ranged tested

NT: Not tested

NA: Not available

CID: PubChem compound identification number

AID: PubChem assay identification number

We found that these inhibitors displayed varying degrees of potency and specificity, which generally agreed with their previously reported activity (Table 1). However, for UM 673A, Pargyline, Daidzin, and CVT-10216, the IC_50_ values obtained in our biochemical assay were 10- to 80-fold higher than their reported IC_50_. In addition, we observed an IC_50_ 15- to 60-fold lower than published for Aldi-2.

We then tested the validation set in the 1,536-well ALDEFLUOR imaging-based assay using both MIA PaCa-2 and HT-29 cells. Eight inhibitors in this validation set (Kyneurine, Citral, Daidzin, DEAB, Disulfiram, Gossypol, Molinate and Pargyline) were recently tested by Marcato and colleagues for their ability to reduce ALDEFLUOR fluorescence in breast cancer cells that express ALDH1A3[43]. The authors found that Citral and DEAB significantly reduced ALDEFLUOR fluorescence (determined by flow cytometry) at concentrations >10 μM. However, it is important to note that most of the inhibitors in the validation set have either not been previously tested or have no reported IC_50_ value using the ALDEFLUOR cellular assay and thus we have no prior data set(s) to compare to. The assay was run according to the protocol described in S1 Table, with each compound tested as a 16-point dilution series, with concentrations ranging from 45.8 μM to 1.4 nM. Compounds displaying high quality concentration response curves and >50% efficacy, were considered active [44]. Under these conditions, 10 compounds exhibited inhibitory activity in both cell lines, with potencies varying from ~0.8 to ~28 μM. The most potent compounds were Compound 5, DEAB, and NCT-501. Importantly, compound potency was similar between cell lines (R^2^ = 0.85, S5B Fig; Table 1). When comparing inhibitor potency between biochemical and cell based-assays we found that compound IC_50_ is higher when inhibitors are tested in the cellular environment as opposed to in the isolated enzyme (Table 1). Only two compounds, Pargyline and Gossypol, were active in HT-29 but inactive in MIA PaCa-2 cells.

Of note is the compound Citral. In the work by Ming et al., the authors utilized Citral as one of the control compounds to validate and optimize different assay parameters such as number of cells/well and substrate removal steps [17]. Under these different conditions, the reported IC_50_ is in the range of ~7.6 to 60.1 μM [17]. In our 1,536-well assay conditions, the IC_50_ for Citral is ~27 μM in both MIA PaCa-2 and HT-29 cells, which agrees with their 96-well assay.

The remaining 8 compounds were inactive in both cell lines. Not surprisingly, compounds like Kynurenine and Molinate, which have low potency (>10 μM) against the purified enzyme also tested negative in our cell-based assays. Other compounds, like CID 2929292, with potent inhibitory activity against isolated ALDH1A1, tested negative in the cellular assay. These results could indicate poor compound cell permeability. Among compounds inactive in the cell-based assay, we also find ALDH3A1-specific inhibitors. Not only do the cell lines tested here not express detectable levels of ALDH3A1 protein (Fig 1A) but the ALDEFLUOR assay reportedly does not detect ALDH3A1 activity[45]. Correspondingly, CB7, a specific and potent inhibitor for ALDH3A1 developed by the Hurley group, tested negative in this cellular assay. Finally, we find that ALDH2-specific inhibitors Daidzin and CVT-10216, also tested negative in the cellular assay. However, as mentioned above, the potency of these two compounds in the *in vitro* assay was in the single and double-digit μM range, respectively, and did not match reported potencies (Table 1). In addition, the observed expression of ALDH2 in both MIA PaCa-2 and HT-29 cells is questionable due to lack of antibody specificity (S1 Fig).

Overall, the results from the biochemical and cellular assays show that Compound 5 and NCT-501 are selective and potent against isolated ALDH1A1 as well as ALDH1A1-expressing cells. Our results identified Disulfiram, Bay 11–7085, Aldi-2, CID 725345, and DEAB as pan-ALDH inhibitors with potent biochemical activity and exhibiting cellular activity in MIAPaCa2 and HT-29 cells.

### Assay automation

**Fig 4:**
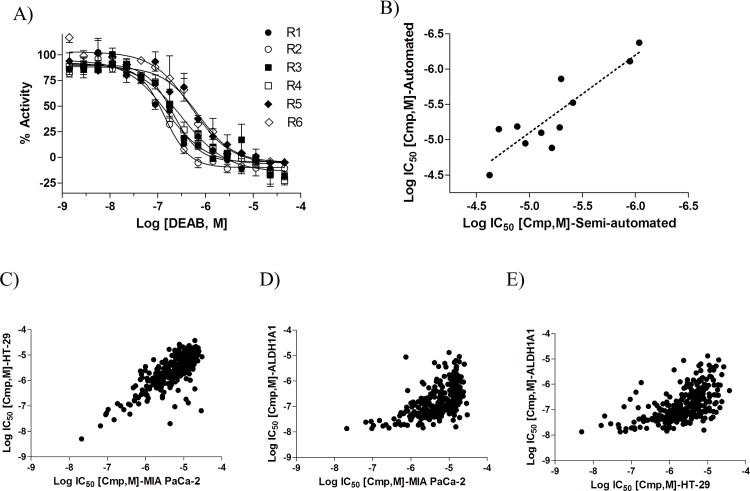
Assay automation. **(A)** DEAB dose response curves of 1,000 cells/well MIA PaCa-2 cells incubated with 500 nM of BAAA substrate for 30 min. R1 to R4 represent four different independent replicates of the fully automated assay. R5 and R6 represent two different independent replicates of the semi-automated assay. Data is represented as mean +/- SD, n = 2. (**B**) Semi- and automated cellular BAA intensity measurements provide similar IC_50_ values for active compounds. Correlation plot of Log IC_50_ values of 11 active compounds in the semi-automated (x-axis) and automated (y-axis) assays. Inactive compounds are not included. (**C**) Correlation plot of Log IC_50_ values of 288 active compounds in MIA-PaCa-2 (x-axis) and HT-29 (y-axis) cells. Inactive compounds are not included. (**D, E**) Correlation plot of Log IC_50_ values for 288 active compounds in enzymatic ALDH1A1 assay (y-axis) and ALDEFLUOR assay (x-axis) in MIA-PaCa-2 (D) and HT-29 (E) cells. Inactive compounds are not included.

In order to support large-scale quantitative screening, we modified the assay protocol to fit our automated robotic platform, containing robotic arms for transporting 1,536-well plates along with dispensers, washers, incubators and detectors. The main difference between the two protocols is the automated removal of media and substrate via aspiration (S1 Table) as opposed to inverting and centrifuging the plate applied in the non-automated screening [25]. Using MIA PaCa-2 cells and following the automated protocol-1 described in S1 Table, the assay yielded a S:B of 3.25±0.64 and Z’ of 0.24±0.03. To determine if the assay statistics could be improved, we added an extra wash step before imaging to further decrease background signal (protocol described in automated protocol-2 inS1 Table). Although this extra step did not alter assay signal window (S:B of 3.5) it did improve assay robustness (Z’ of 0.53±0.05). We then screened the validation set using the above fully automated protocol-2 with two washes and compared inhibitor activity with that obtained using the semi-automated format described in S1 Table. The IC_50_ for control DEAB was similar in both the semi- and fully automated formats (Fig 4A; IC_50_ range of 0.14 to 0.70 μM). Almost all compounds that showed no inhibitory activity in the semi-automated assay, also showed no activity in the fully automated format. The only discrepancy was Pargyline, which exhibited no activity in the semi-automated protocol but reduced BAA signal with an IC_50_ of ~5 μM in the automated protocol. Potencies for the remaining 11 compounds with BAA-reducing activity correlated well (R^2^ = 0.78, p<0.0002) between protocol formats (Fig 4B).

To demonstrate the power of this automated cellular assay, we tested a collection of 379 ALDH1A1 inhibitors developed as part of an in-house campaign (manuscript in preparation). This collection contains inhibitors with potencies ranging from 13 nM to 14 μM activity in ALDH1A1 biochemical assays. We tested each compound as an 11-point, 1:3 dilution series (final concentration range 0.775 nM to 45.8 μM) against both MIA PaCa-2 and HT-29 cells. A total of 288 compounds demonstrated inhibitory activity in both cell lines and with IC_50_ values ranging from 0.02 to 30 μM (high quality concentration response curves and >50% efficacy). Inhibitor IC_50_ values correlated well in both cell lines with an R^2^ = 0.57 and p<0.0001 (Fig 4C). When comparing IC_50_ values between ALDH1A1 enzymatic and cell-based assays, similar to the validation set, compound potency is right-shifted in the cell-based assay (Fig 4D and 4E, R^2^ values of 0.31 and 0.33, respectively). The median potency difference between biochemical and MIA PaCa-2 and HT-29 assays were 38- and 17-fold, respectively. Of the remaining 91 compounds, 24 reduced BAA intensity in only one of the cell lines, and 67 compounds did not reduce BAA intensity in either of the cell lines tested. Importantly, these inactive compounds showed potency ranges of 0.07 to 34.6 μM in biochemical settings, indicating that our assay identifies analogs with good biochemical activity but poor cell permeability and/or intracellular target engagement.

### The 1,536-well high-content ALDEFLUOR assay detects ALDH1A1-specific inhibitors

**Fig 5:**
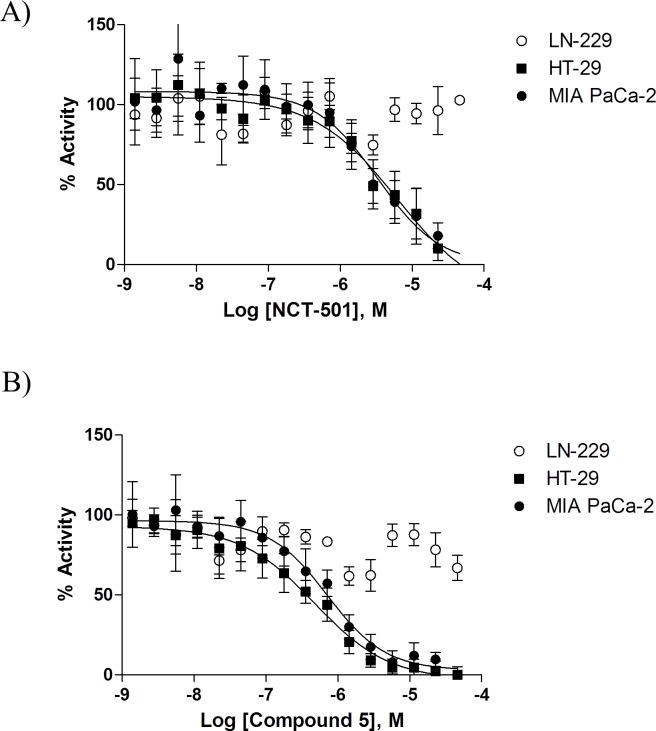
Differential ALDH-expressing patterns allow the detection of specific ALDH1A1 inhibitors. Dose response curves of ALDH1A1-specific inhibitors NCT-501 (A) and Compound 5 (B) in MIA PaCa-2 and HT-29 (ALDH1A1-positive cells) vs. LN-229 (ALDH1A1-negative cells). The IC_50_ for NCT-501 and Compound 5 is 6.26 and 0.71 μM in MIAPaCa2 cells and 3.3 and 0.47 μM in HT-29 cells. Data are represented as mean +/- SD, n = 4.

We next sought to test if the fully automated high-content assay could be used to detect ALDH1A1-specific inhibitors. To this end, we compared the activity of control DEAB and the ALDH1A1-specific inhibitors NCT-501 and Compound 5 in cells with high (MIAPaCa2 and HT-29) versus low (LN-229) ALDH1A1 protein levels. Importantly, LN-229 displays detectable levels of BAA fluorescence, likely due to high expression levels of ALDH1A3 and possibly ALDH2 (Fig 1A and S6A Fig). Under the same conditions tested for MIA PaCa-2 and HT-29 (1,000 cells/well, 500 nM substrate and an incubation time of 30 minutes), LN-229 cells yielded an IC_50_ of ~3.48 μM for control DEAB and the assay exhibited a S:B ~1.7 (S6B Fig). As shown in Fig 5, NCT-501 and Compound 5 inhibited BAAA turnover only in ALDH1A1-expressing cells.

### A dual cell-based screen identifies ALDH1A1-specific inhibitors

The above findings prompted us to speculate that a dual cell-based screening paradigm using a pair of cell lines with high and low ALDH1A1 expression could be implemented as a phenotypic imaging-based screen to identify novel ALDH1A1 inhibitors from large compound collections. We reasoned that compounds that reduce BAA intensity exclusively in MIA PaCa-2 cells but are inactive in LN-229 cells, are ALDH1 inhibitors specific for the 1A1 isozyme. This paradigm would directly identify cell-permeable inhibitors, in a process that bypasses the initial biochemical screen and subsequent counterscreens for target specificity.

**Fig 6:**
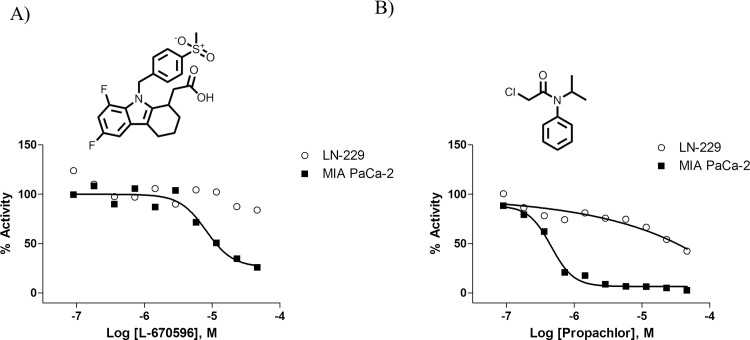
A dual cell-based screening paradigm allows the identification of novel compounds with ALDH1A1 specific inhibitory activity. Structure and representative dose response curves of L-670596 (**A**) and Propachlor (**B**) in MIA PaCa-2 (ALDH1A1-positive cells) and LN-229 (ALDH1A1-negative cells).

To validate our hypothesis, we screened a collection of bioactive molecules containing 1,279 unique compounds in dose response (7 concentration points ranging from 2.9 nM to 45.8 μM; LN-229 cells yielded a S:B = 2.3 and Z’ = 0.22; MIA PaCa-2 cells yielded a S:B = 3.5 and Z’ = 0.36) following the automated protocol-1 in S1 Table (no extra wash step included). A total of 6 compounds reduced BAA activity in MIA PaCa-2 but not in LN-229. We were able to source 5 of the 6 compounds for retesting, at an 11-point dose response with a concentration range of 0.77 nM to 45.8 μM. Among these, we confirmed the MIA PaCa-2-specific activity of the thromboxane receptor antagonist L-670596 (PubChem CID 129360) with an IC_50_ of ~10 μM (Fig 6A and Table 2). The herbicide Propachlor (PubChem CID 4931), showed a >50-fold difference in potency between MIA PaCa2 and LN-229 cells (IC_50_ of 0.3 vs. 16 μM, respectively; Fig 6B and Table 2). The remaining three compounds retested were either inactive or showed no potency difference between cell lines. *In vitro* enzymatic assays validated L-670596 as a specific ALDH1A1 inhibitor. Similarly, Proprachlor is a much more potent inhibitor of ALDH1A1 vs. the other isozymes tested (Table 2). Neither Proprachlor nor L-670596 have previously been shown to possess anti-aldehyde hydrogenase activity.

**Table 2:** IC_50_ values of ALDH1A1-selective compounds identified from dual cell-based screen.

		Biochemical IC_50_ [μM]					ALDEFLUOR IC_50_ [μM]	
Sample Name	PubChem SID	ALDH1A1	ALDH1A2	ALDH1A3	ALDH2	ALDH3A1	MIA PaCa2	LN229
L-670596	11114241	0.25	ND	35.48	ND	ND	10.75	ND
Propachlor	26747749	0.01	0.32	5.62	12.59	50.12	0.30	16.10

ND: Not determined in the concentration ranged tested.

SID: PubChem substance identification number.

## Discussion

Here we developed and demonstrated a fully automated 1,536-well high-content ALDEFLUOR assay that can be scaled to a robotic screening platform to evaluate large compound libraries and in high-density dose-response (i.e. 7 or 11-points). The imaging time required per plate is ~20 min. By fully automating the assay, where the only user intervention is supplying plated cells, one can run up to 72 plates (or ~10,000 compounds at 11-point dose response) per day when only one plate reader is used. The high-throughput offered by our assay is especially advantageous when using low-abundant cells, such as patient-derived cells. With similar number of cells needed per plate format (9.6 x 10^5^ vs. 1.5x10^6^ for 96- and 1,536-well format, respectively), one can screen 17 times more compounds at 11-point dose range in the 1,536-well format compared to the earlier 96-well format assay[17]. In addition, our miniaturized format is cost-effective in terms of compound volumes and ALDEFLUOR reagent usage, since it allows one to screen 7 times more compounds with the same amount of reagent used in the 96-well format.

We implemented our high-content assay to quantitatively benchmark the potential of prior art compounds to reduce ALDH1A1 activity in a cellular context. To our knowledge, this is the first study in which a comprehensive set of ALDH inhibitors is tested in parallel in both biochemical and cell-based assays. Moreover, our assay provides the throughput necessary to support medicinal chemistry efforts during lead optimization processes. For some of the prior art compounds, we observed IC_50_ values that were higher or lower than previously published results, which could be attributed to differences in assay format, buffer components, reaction progress, substrate concentration, and/or compound quality (i.e. purity).

In drug discovery programs, a frequent practice consists of first assaying compounds in a biochemical assay and then advancing selected molecules to biochemical counter-assays (to assess target specificity) as well as to a cellular format to identify cell-permeable structures. The dual cell-based screening paradigm presented here allows the direct identification of ALDH1A1-specific (cell type-specific) inhibitors with cellular permeability and potency. This set-up offers an attractive alternative approach to the discovery of ALDH1A1 inhibitors as it enables one to expedite the identification of cellular active lead compounds from large collections.

The assay presented herein represents an important advance for expediting ALDH1A1 drug discovery campaigns, complementing *in vitro* biochemical assays, providing the throughput necessary to support SAR efforts and also offering the alternative to be used as a primary phenotypic screen to discover ALDH1A1 inhibitors from large collections.

## Materials and Methods

### Cell lines and culture conditions

MIA PaCa-2, PANC-1, HT-29, SW480, LN-18 and LN-229 cells were obtained from America Type Culture Collection, (ATCC, Manassas, VA; #CRL-1420, CRL-1469, HTB-38, CCL-228, CRL-2610 and CRL-2611, respectively). MIA PaCa-2, PANC-1, HT-29 and SW480 were cultured in RPMI 1640 (Life Technologies, Carlsbad, CA), supplemented with 2mM L-Glutamine (Life Technologies), 10% HyClone fetal bovine serum (FBS, GE Healthcare, Piscataway, NJ) and 100 U/mL penicillin and 100 μg/mL streptomycin (referred to as 1% Pen/Strep, Life Technologies). PANC-1, LN-18 and LN-229 were cultured in DMEM (Life Technologies) supplemented with 10% FBS and 1% Pen/Strep. All cell lines were maintained at 37°C, 5% CO_2_, 85% RH,routinely tested for mycoplasma contamination and authenticated by short tandem repeat (STR) profiling.

### Compounds

DEAB, Pargyline, and Aldi-2 were obtained from Sigma-Aldrich (St. Louis, MO). Daidzin, Compound 5, and A37 were obtained from Biomol (Hamburg, Germany), Chemdiv (San Diego, CA), and Glixx (Southborough, MA), respectively. UM 673A and UM 673B were sourced from ChemBridge (San Diego, CA). Molinate and Citral were obtained from NIEHS (Durham, NC). CVT-10216 and Bay-11-7085 were sourced from Tocris (Bristol, UK). Disulfiram, Kynurenine, Gossypol, and Nitroglycerin were purchased from Microsource (Gaylordsville, CT). CID 2929292, CID 725345, NCT-501, and indoline-2,3-dione were synthesized in-house. All compounds were subjected to quality control by LC/UV, LC/MS, or Hi-res MS. All compounds exhibited >90% purity by peak area or *m/z* with the exception of Citral, CID 725345 and CB7, which had 60–90% purity but identity was confirmed.

### Western blot

Whole cell lysates were prepared using RIPA buffer (Cell Signaling Technologies, Danvers, MA) and protease inhibitor cocktail (Sigma-Aldrich). Cell lysates were quantified using the Bio-Rad DC Protein Assay (Bio-Rad, Hercules, CA). Eighteen μg of protein or 50 ng of indicated recombinant ALDH were loaded on 4–12% gradient NuPAGE Novex Bis-Tris gels (ThermoFisher, Carlsbad, CA) in MES SDS running buffer. Proteins were transferred to nitrocellulose membranes using the iBlot 2 Gel Transfer Device (ThermoFisher) and blocked in 5% blotting milk in TBST buffer (50 mM Tris pH 7.5, 150 mM NaCl, 0.05% Tween20). Membranes were incubated with either mouse anti-ALDH1 (BD Biosciences #611194, San Jose, CA), rabbit anti-ALDH1A2 (Abcam #ab156019, Cambridge, MA), rabbit anti-ALDH1A3 (Abcam #ab129815), rabbit anti-ALDH2 (Abcam #ab108306) or rabbit anti-ALDH3A1 (Abcam #ab129022) at 1:1,000 dilution and rabbit anti-β-Actin primary antibodies (Cell Signaling Technologies # 4970L) primary antibodies at 1:5,000 dilution overnight (4°C). HRP-conjugated secondary antibodies were used as follow: HRP-anti-rabbit IgG (Cell Signaling Technologies #7074S) or HRP-anti-mouse IgG (Cell Signaling Technologies #7076S) at 1:10,000 dilution, incubated (RT) for 1 hour, and visualized with SuperSignal West Dura Chemiluminescent Substrate (ThermoFisher) on a Bio-Rad Gel Doc XR+ Gel Documentation System.

### 1,536-well enzymatic assays

Human ALDH1A1 and ALDH3A1 were expressed and purified as described elsewhere [46, 47]. Human ALDH2 was purchased from Abcam (ab87415). Human ALDH1A2 and ALDH1A3 were purchased from MyBioSource (MBS1005929; San Diego, CA) and ThermoFisher (11636H07E50), respectively. The inhibitory activity of compounds against ALDHs was measured according to protocols described previously [28]. Briefly, 3 μL of enzyme (final concentrations of 20 nM for ALDH1A1, 50 nM for ALDH1A2, 50 nM for ALDH1A3, 75 nM for ALDH2 and 5 nM for ALDH3A1) in assay buffer (100 mM HEPES pH 7.5 with 0.01% Tween 20) were dispensed into a 1,536-well solid-bottom black plate (Greiner Bio One, Monroe, NC). Twenty-three nL of compounds (final concentration range 968 pM to 57.2 μM) or control Bay 11–7085 (final concentration range 1.31 nM to 2.86 μM) were transferred via Wako Pin-tool (Wako Automation, Richmond, VA). Samples were incubated (room temperature, protected from light) for 15 minutes followed by a 1 μL substrate addition of NAD^+^ and Propionaldehyde (final concentrations of 1 mM and 80 μM, respectively) for ALDH1A1, 1A2, 1A3, and ALDH2 or NADP^+^ and benzaldehyde (final concentrations of 1 mM and 200 μM, respectively) for ALDH3A1. Plates were centrifuged at 1,000 rpm for 15 seconds, then read in kinetic mode on a ViewLux High-throughput CCD imager (PerkinElmer, Waltham, MA) equipped with standard UV fluorescence optics (340 nm excitation, 450 nm emission) for 5 (ALDH1A1, ALDH1A2, ALDH2, ALDH3A1) or 30 (ALDH1A3) minutes. The change in fluorescence intensity over the 5- or 30-minute reaction period was normalized against no-inhibitor and no-enzyme controls and the resulting percent inhibition data were fitted to a 4-parameter Hill equation. Bay-11 7085 was used as intraplate control. To account for artifacts due to intrinsic compound fluorescence in the above spectrum, compounds were also tested in an orthogonal resorufin assay format with optics of excitation 525 nm/emission 598 nm as described before[29]. The same parameters as the NADH assay were used for the resorufin assay, with the addition of (final) 133 μg/mL diaphorase and 37 μM resazurin to the assay solution. Same controls were used in both formats.

### 1,536-well high-content imaging ALDEFLUOR assay

The ALDEFLUOR kit was purchased from STEMCELL Technologies (Vancouver, Canada; #01700). Cells (5 μL) were dispensed into black, optical quality (cyclic olefin copolymer) clear bottom, medium binding TC treated 1,536-well plates (Aurora Microplates, Whitefish MT) using a Multidrop Combi dispenser (ThermoFisher) and incubated overnight (37°C, 5% CO_2_, 85% RH). Media was subsequently removed by centrifuging plates upside down using a plate adaptor to collect media. A solution of BAAA substrate (STEMCELL Technologies) and Hoechst 33342 (ThermoFisher, final concentrations of 500 nM and 0.5 nM, respectively) in ALDEFLUOR buffer (STEMCELL Technologies #01700) was dispensed onto cells using a Multidrop Combi followed by immediate transfer (23 nL) of compound or control solutions using a Wako Pin-tool (final percentage of DMSO in the cell plates was 0.5%). Unless otherwise noted, all compounds were assayed as 16-point dilutions spanning a final concentration range of 1.4 nM to 47.8 μM. The neutral and positive assay controls were DMSO and DEAB (4.6 μM), respectively. Cells were incubated for 30 minutes or the indicated amount of time at 37°C, 5% CO_2_, 85% RH to allow the conversion of BAAA into BAA. Supernatant was subsequently removed by centrifugation as described above, then ALDEFLUOR buffer (3 μl) was dispensed by Multidrop Combi before imaging on an IN Cell 2200 (GE Healthcare).

The above assay was modified for an online robotic screening system. After cell plating and overnight incubation, 4 μL of media were removed using a 64-tip metal aspirator head on a Wako aspirator station, leaving 1 μL remaining in the well, followed by a 4 μL dispense of BAAA and Hoechst 33342 in ALDEFLUOR buffer, for a final concentration of 500 nM and 0.5 nM, respectively. Immediately following the dispense, 23 nL of compound or control solutions were transferred using a Wako Pin-tool. Cells were incubated for 30 minutes at 37°C, 5% CO_2_, 85% RH, followed by a 4 μL media removal using the Wako aspirator, and a subsequent 3 μL addition of ALDEFLUOR buffer. Plates were then immediately read on the IN Cell 2200 as described below.

### Image acquisition and analysis

For images captured on the IN Cell 2200 widefield automated microscope, a 10x 0.45 NA Plan Apo objective lens was used to capture the entire well of the 1,536-well plate using standard DAPI (390/18x, 432/48m) and FITC (475/28x, 525/48m) filter sets at 50 msec and 100 msec exposures, respectively. Images were subsequently analyzed using IN Cell Investigator v1.6.2 analysis software’s canned Multi-Target Analysis algorithm (GE Healthcare). Hoechst stained nuclei were identified using top hat segmentation with a minimum area of 75 μm^2^ and sensitivity of 93. BAA-retaining cells captured via FITC channel, were identified using multiscale top hat segmentation with a minimum area of 100 μm^2^ and a sensitivity setting of 16. Several data measures were collected and the most robust measure for ALDH activity was found to be the integrated intensity (Intensity x Area) of the FITC channel. Data were plotted using GraphPad Prism software (GraphPad, San Diego, CA), with sigmoidal dose-response (variable slope) fitting.

### qHTS data analysis and statistics

Data from each assay were normalized plate-wise to corresponding intra-plate controls (neutral control DMSO and positive control DEAB 4.6 μM or otherwise noted) as described previously[48]. The same controls were also used for the calculation of the Z’ factor for each assay. The Z’ factor, a measure of assay quality control, was determined as previously described[49]. Percent activity was derived using in‐house software[50]. Dose-response curves were classified as described previously[44]. All concentration–response curves were fitted as before and IC_50_ were calculated with the GraphPad Prism software.

## Supporting Information

S1 Fig Antibody specificity analysis. (TIF)

S2 Fig Fluorescence intensity correlates to ALDH1A1 and substrate levels. (TIF)

S3 Fig Removing remaining BAAA before imaging improves assay signal window and robustness. (TIF)

S4 Fig Structure of compounds in the validation set. (TIF)

S5 Fig Determination of IC_50_ values for validation set compounds correlates well between assay formats. (TIF)

S6 Fig ALDEFLUOR imaging assay in LN-229 cells. (TIF)

S1 Table Protocols for 1,536-well ALDEFLUOR assay. (DOCX)

## Abbreviations

ALDH: aldehyde dehydrogenase
qHTS: quantitative high-throughput screening
NAD(P)+: Nicotinamide adenine dinucleotide (phosphate)
BAAA: BODIPY-aminoacetaldehyde
BAA: BODIPY-aminoacetate
DMSO: dimethyl sulfoxide
DEAB: 4-N,N-diethylaminobenzaldehyde
