# Correction: A High-Content Assay Enables the Automated Screening and Identification of Small Molecules with Specific ALDH1A1-Inhibitory Activity

**DOI:** 10.1371/journal.pone.0197292

**Published:** 2018-05-15

**Authors:** Adam Yasgar, Steven A. Titus, Yuhong Wang, Carina Danchik, Shyh-Ming Yang, Vasilis Vasiliou, Ajit Jadhav, David J. Maloney, Anton Simeonov, Natalia J. Martinez

S5 Fig is incorrect. The correct S5 Fig can be viewed below.

## Supporting information

S5 Fig Determination of IC_50_ values for validation set compounds correlates well between assay formats. (TIF)
