# Supplementary figures and images for: A High-Content Assay Enables the Automated Screening and Identification of Small Molecules with Specific ALDH1A1-Inhibitory Activity
Source: PLoS One. 2017 Jan 27;12(1):e0170937. doi: 10.1371/journal.pone.0170937 (PMC5271370; doi:10.1371/journal.pone.0170937)

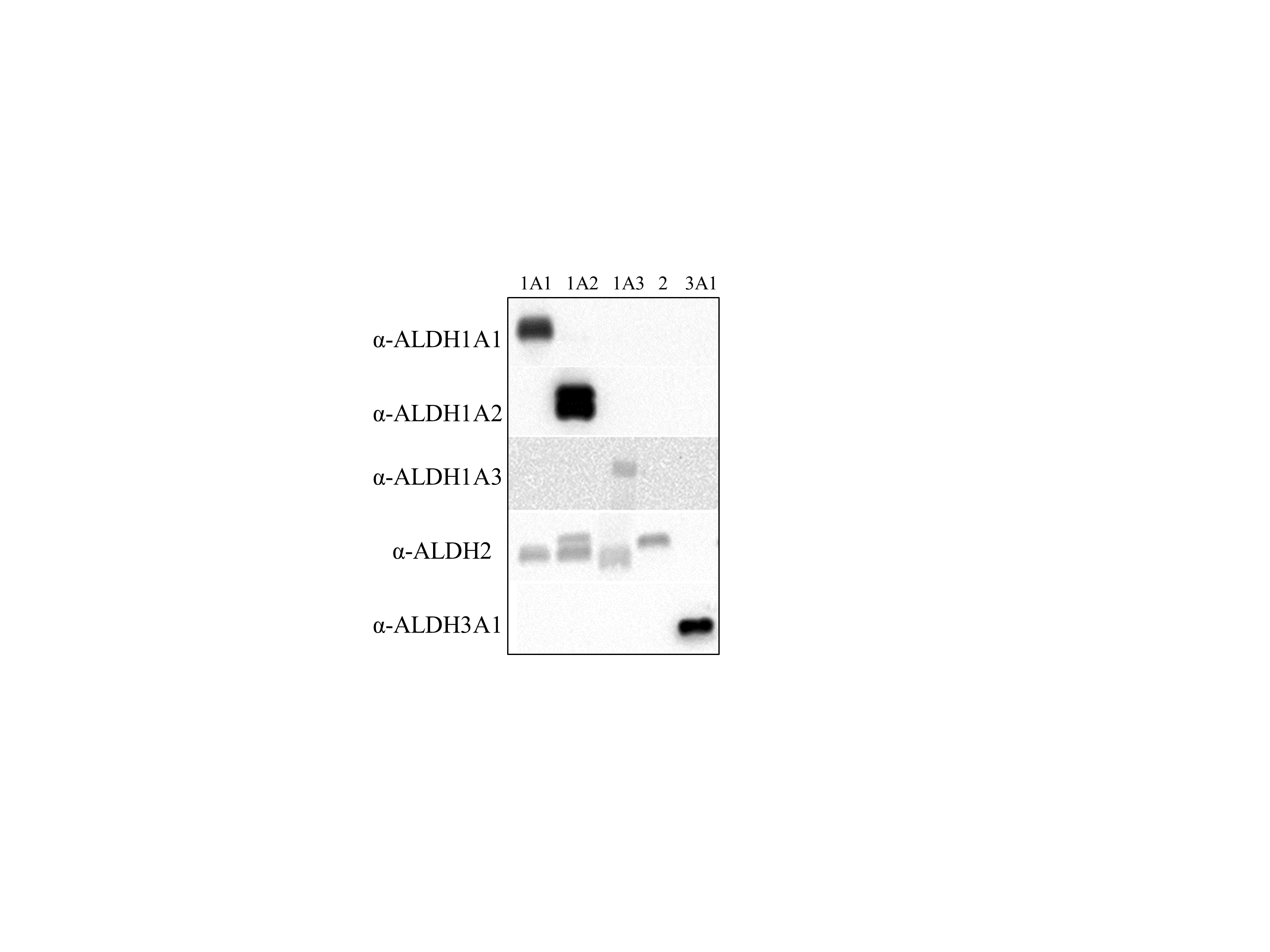

Supplement: S1 Fig — (TIF) [file pone.0170937.s001.TIF]

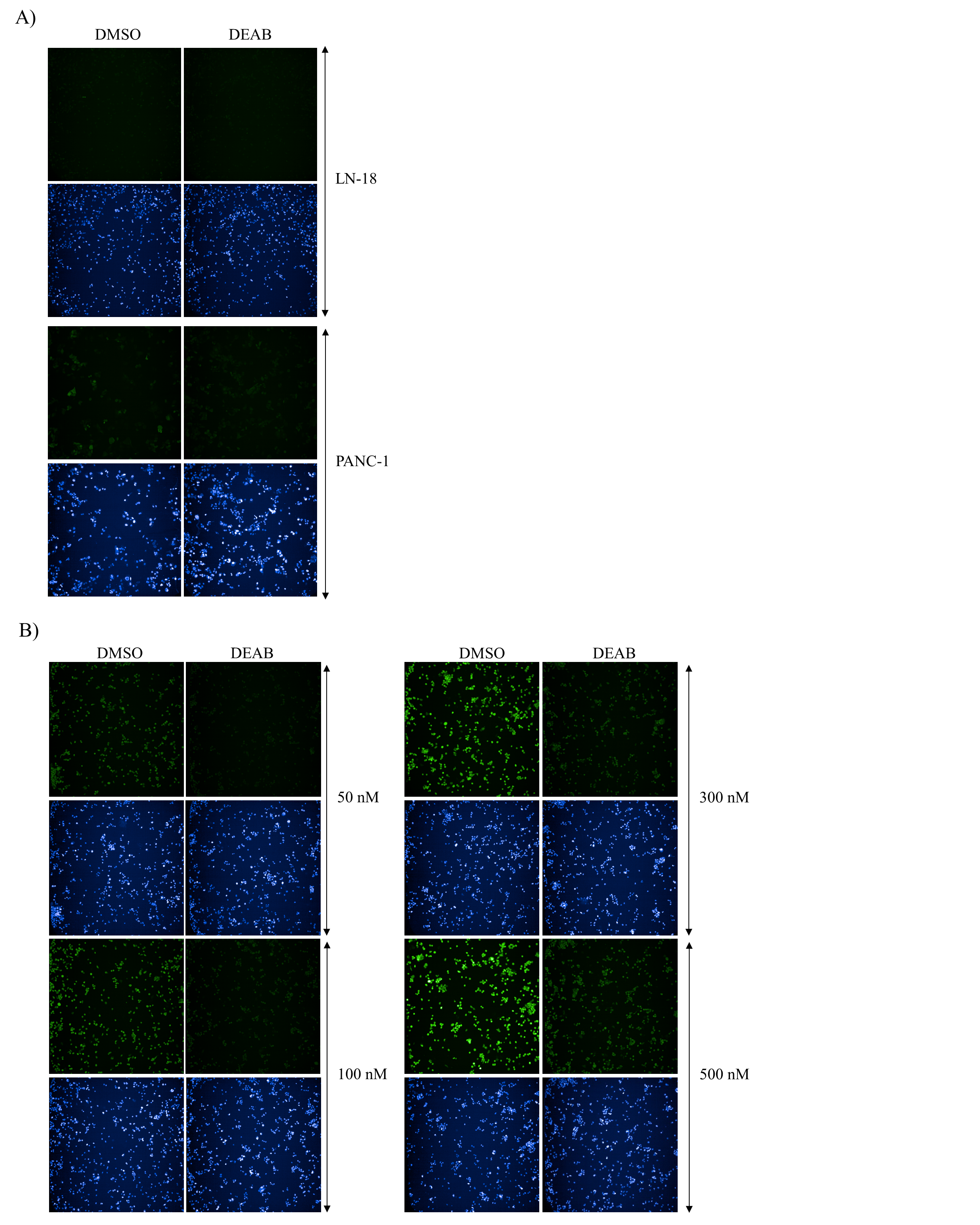

Supplement: S2 Fig — (TIF) [file pone.0170937.s002.tif]

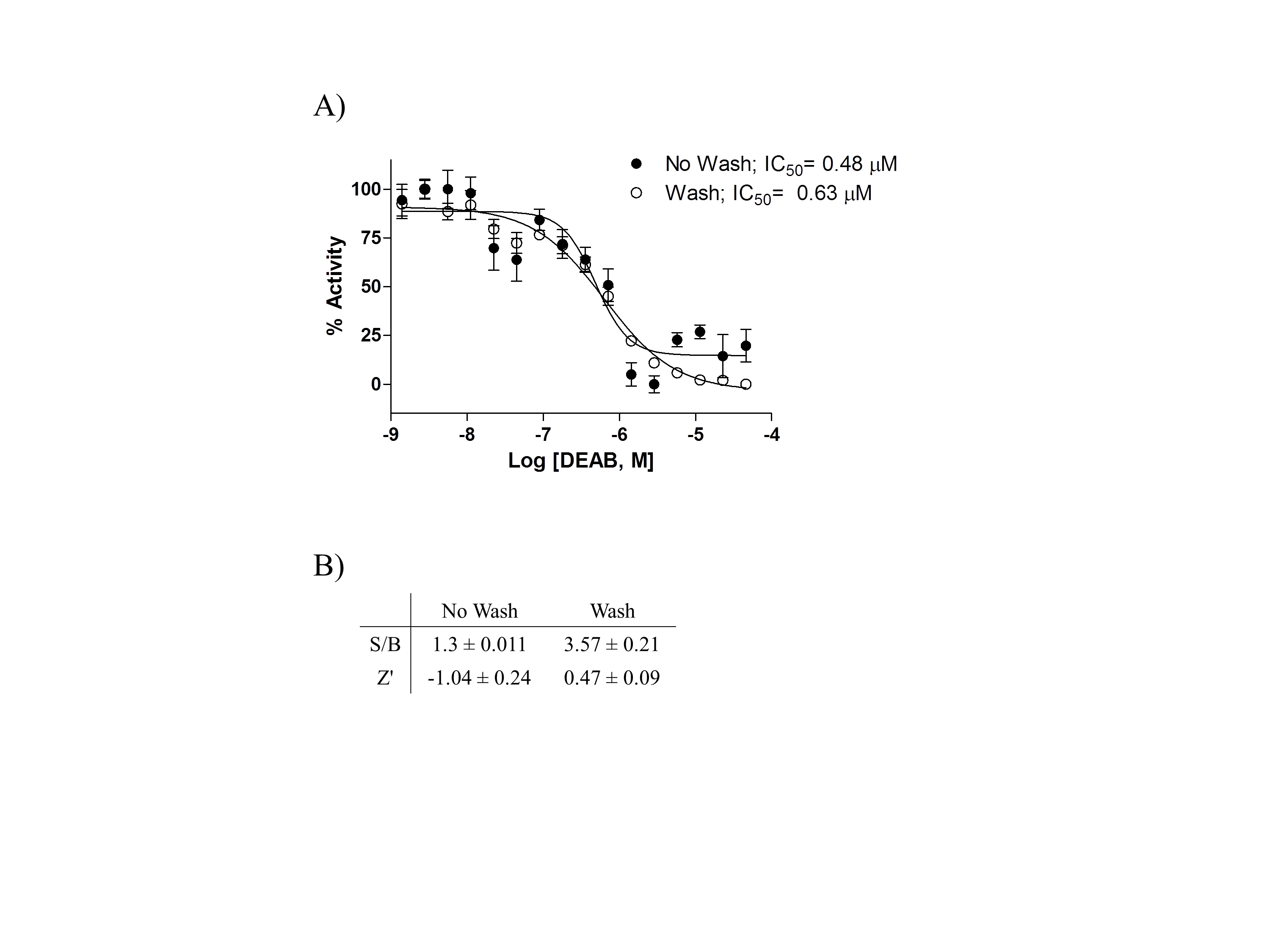

Supplement: S3 Fig — (TIF) [file pone.0170937.s003.TIF]

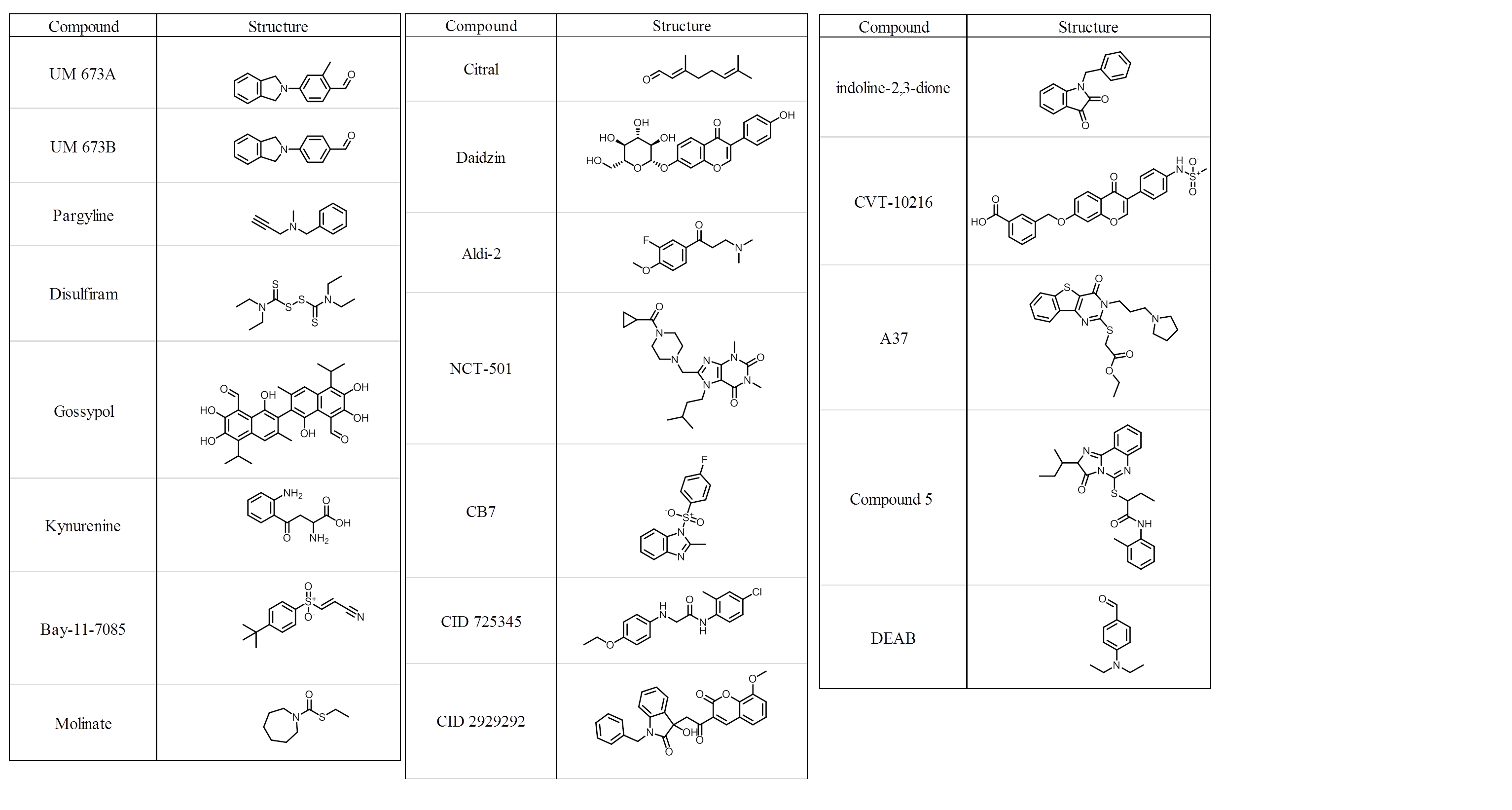

Supplement: S4 Fig — (TIF) [file pone.0170937.s004.TIF]

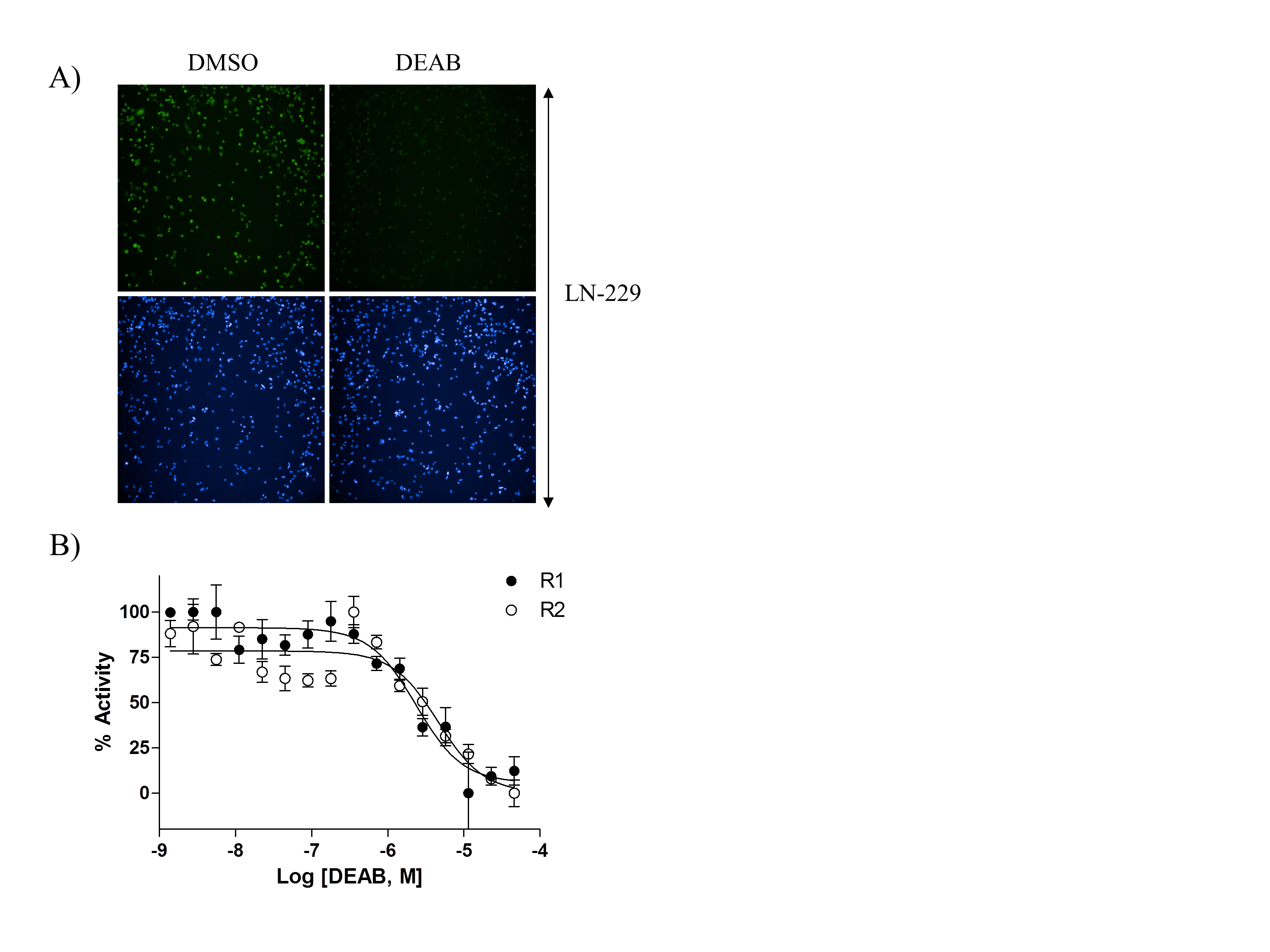

Supplement: S5 Fig — (TIF) [file pone.0170937.s005.tif]

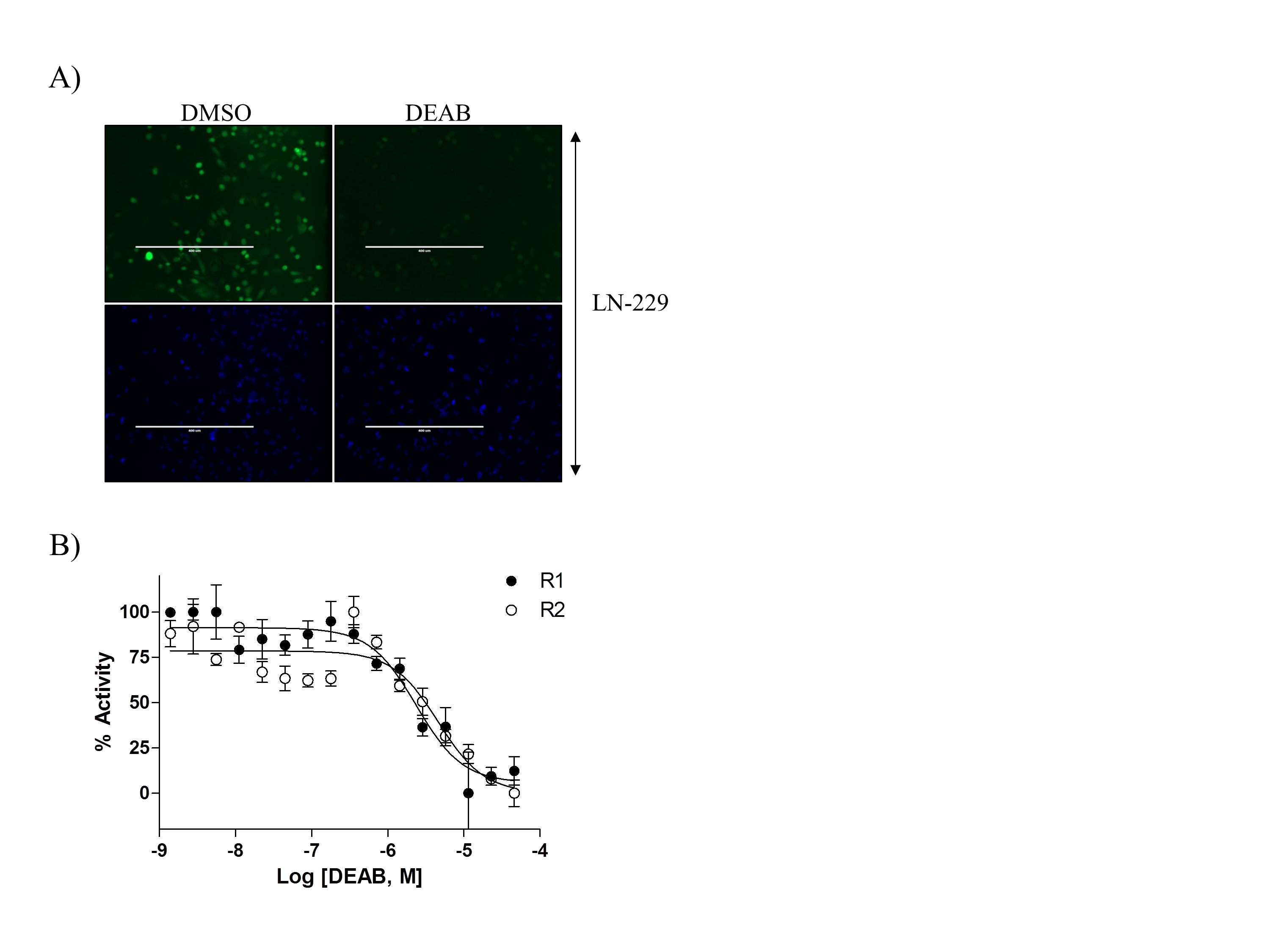

Supplement: S6 Fig — (TIF) [file pone.0170937.s006.TIF]
