# Supplementary material for: A High-Content Assay Enables the Automated Screening and Identification of Small Molecules with Specific ALDH1A1-Inhibitory Activity
Source: PLoS One. 2017 Jan 27;12(1):e0170937. doi: 10.1371/journal.pone.0170937 (PMC5271370; doi:10.1371/journal.pone.0170937)
Supplement: S1 Table — (DOCX) [file pone.0170937.s007.docx]

**Supplementary Information**

**A high-content assay enables the automated screening and identification of small molecules with specific ALDH1A1-inhibitory activity**

Adam Yasgar*^1^*, Steven A. Titus*^1^*, Yuhong Wang*^1^*, Carina Danchik*^2^*, Shyh-Ming Yang*^1^*, Vasilis Vasiliou*^2^*, Ajit Jadhav*^1^*, David J. Maloney*^1^*, Anton Simeonov*^1*^* and Natalia J. Martinez*^1*^*

*^1^* National Center for Advancing Translational Sciences, National Institutes of Health, Rockville, MD, United States of America.

*^2^*Department of Environmental Health Sciences, Yale School of Public Health, New Haven, CT, United States of America.

*Corresponding authors [asimeono@mail.nih.gov](mailto:asimeono@mail.nih.gov) (AS); [natalia.martinez@nih.gov](mailto:natalia.martinez@nih.gov) (NJM)

This file includes:

Supplementary Figures S1-S6 legends

Supplementary Tables 1

*Supplementary Figure Legends*

**S1 Fig. Antibody specificity analysis.** Western blot analysis of ALDH1A1, ALDH1A2, ALDH1A3, ALDH2 and ALDH3A1 antibodies against the panel of recombinant proteins.

**S2 Fig. Fluorescence intensity correlates to ALDH1A1 and substrate levels.** (**A**) ALDH1A1-low expressing cells have undetectable levels of BAA. Representative fluorescence images of LN-18 and PANC-1 cells incubated with 500 nM of BAAA substrate and treated with DMSO vehicle for 30 minutes. The green images (top) indicate lack of intracellular BAA, while Hoechst stained nuclei (blue images) indicate total number of cells in the well (bottom). (**B**) Increasing amount of BAAA substrate leads to increase fluorescence intensity. Representative fluorescent images of MIA PaCa-2 cells incubated with 500, 300, 100 ornM of BAAA substrate and treated with DMSO vehicle for 30 minutes. The green images (left panels) indicate levels of intracellular BAA, while blue images (right panels) indicate total number of cells in the well.

**S3 Fig. Removing remaining BAAA before imaging improves assay signal window and robustness. (A)** DEAB dose response curves of 2,000 MIA PaCa-2 cells/well incubated with 100nM BAAA substrate for 30 min. Imaging was performed before (No Wash) or after removing (Wash) remaining BAAA substrate. IC_50_ are indicated for both conditions. **(B)** Washing step improves assay S/B and Z’.

**S4 Fig. Structure of compounds in the validation set.**

**S5 Fig. Determination of IC_50_ values for validation set compounds correlates well between assay formats. (A)** Correlation plot of Log IC_50_ values for validation set compounds in enzymatic ALDH1A1 assay run using a NADH-based format (x-axis) and resorufin-based format (y-axis). (**B**) Correlation plot of Log IC_50_ values for validation set compounds in cell-based imaging assay run using HT-29 (x-axis) and MIA PaCa-2 (y-axis) cells.

**S6 Fig. ALDEFLUOR imaging assay in LN-229 cells.** (**A**) LN-229 cells have detectable levels of BAA. Representative fluorescent images of cells incubated with 500 nM of BAAA substrate and treated with DEAB or DMSO vehicle for 30 minutes. The green images (top) indicate intracellular BAA, while blue images (bottom) indicate total number of cells in the well. **(B)** DEAB dose response curves of 1,000 LN-229 cells/well incubated with 500 nM of BAAA substrate for 30 min. R1 and R2 represent independent replicates.

*Supplementary Table*

**S1 Table. Protocols for 1,536-well ALDEFLUOR assay**

|  | | | **Protocol** | | |
| --- | --- | --- | --- | --- | --- |
|  | | | **Semi-automated** | **Automated protocol 1** | **Automated protocol 2** |
| **Step** | **Parameter** | **Value** | **Description** | **Description** | **Description** |
| **1** | Plate cells | 5 μL | 1,000 cells/well are dispensed into 1536-well, low base, black clear bottom plates. Incubate 5-16 h at 37ºC, 5% CO_2_ | 1,000 cells/well are dispensed into 1536-well, low base, black clear bottom plates. Incubate 5-16 h at 37ºC, 5% CO2 | 1,000 cells/well are dispensed into to1536-well, low base, black clear bottom plates. Incubate 5-16 h at 37ºC, 5% CO_2_ |
| **2** | Remove media | 5 μL | Plate is inverted and centrifuge for 15 seconds at 1,000 rpm to remove all media | Aspirate media leaving 1 μL in the well | Aspirate media leaving 1 μL in the well |
| **3** | Reagent Dispense | 5 μL | Dissolve BAAA and Hoechst 33342 nuclear dye in ALDEFLUOR buffer (for final 500 nM and 0.5 nM, respectively) | Dissolve BAAA and Hoechst 33342 nuclear dye in ALDEFLUOR buffer (for final 500 nM and 0.5 nM, respectively) | Dissolve BAAA and Hoechst 33342 nuclear dye in ALDEFLUOR buffer (for final 500 nM and 0.5 nM, respectively) |
| **4** | Compound addition | 23 nL | Pintool transfer; | Pintool transfer | Pintool transfer |
| **5** | Incubation | 30 min | 37ºC, 5% CO_2_, 85% Rh | 37ºC, 5% CO_2_, 85% Rh | 37ºC, 5% CO_2_, 85% Rh |
| **6** | Wash | 5 μL | Plate is inverted and centrifuge for 15 seconds at 1,000 rpm to remove all reagent | Aspirate media leaving 1 μL in the well | Aspirate media leaving 1 μL in the well;  Dispense µL ALDEFLUOR buffer and aspirate leaving 1 μL in the well |
| **7** | Reagent Dispense | 3 μL | ALDEFLUOR buffer | ALDEFLUOR buffer | ALDEFLUOR buffer |
| **8** | Assay readout | DAPI and FITC filter sets | IN Cell 2200 | IN Cell 2200 | IN Cell 2200 |
