# Supplementary figures and images for: Correction: A High-Content Assay Enables the Automated Screening and Identification of Small Molecules with Specific ALDH1A1-Inhibitory Activity
Source: PLoS One. 2018 May 15;13(5):e0197292. doi: 10.1371/journal.pone.0197292 (PMC5953492; doi:10.1371/journal.pone.0197292)

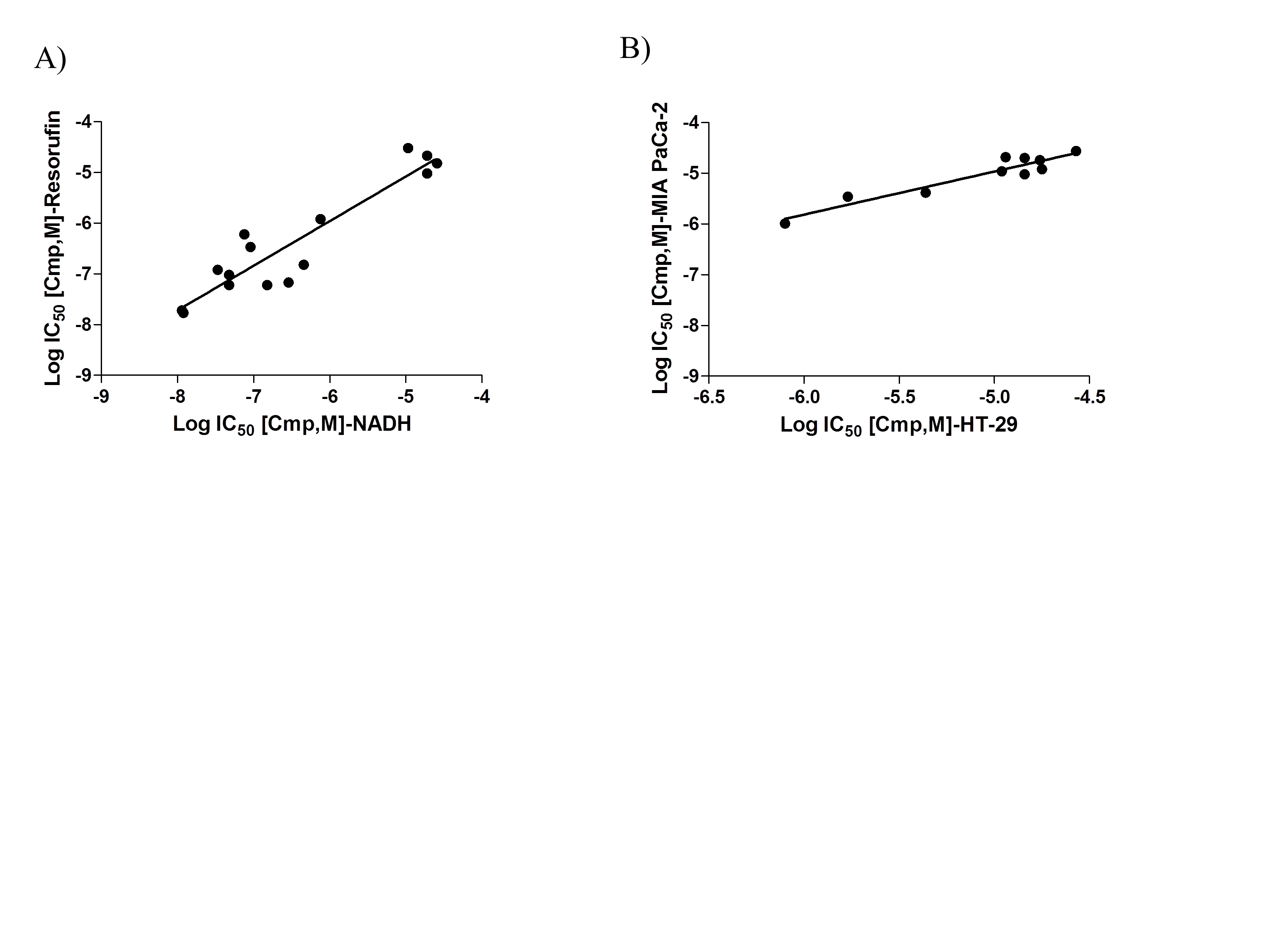

Supplement: S5 Fig — (TIF) [file pone.0197292.s001.TIF]
